# Diffusion-weighted magnetic resonance imaging of primary cervical cancer in the detection of sub-centimetre metastatic lymph nodes

**DOI:** 10.1186/s40644-020-00303-4

**Published:** 2020-04-06

**Authors:** Jose Angelo Udal Perucho, Keith Wan Hang Chiu, Esther Man Fung Wong, Ka Yu Tse, Mandy Man Yee Chu, Lawrence Wing Chi Chan, Herbert Pang, Pek-Lan Khong, Elaine Yuen Phin Lee

**Affiliations:** 1 Department of Diagnostic Radiology, Li Ka Shing Faculty of Medicine, The University of Hong Kong, Room 406, Block K, Queen Mary Hospital, Pok Fu Lam Road, Pok Fu Lam, Hong Kong; 2 Department of Radiology, Pamela Youde Nethersole Eastern Hospital, 3 Lok Man Road, Chai Wan, Hong Kong; 3 Department of Obstetrics and Gynaecology, Li Ka Shing Faculty of Medicine, The University of Hong Kong, 6/F, Professorial Block, Queen Mary Hospital, Pok Fu Lam Road, Pok Fu Lam, Hong Kong; 4 Department of Health Technology and Informatics, Hong Kong Polytechnic University, Room Y934, 9/F, Lee Shau Kee Building, The Hong Kong Polytechnic University, Hung Hom, Hong Kong; 5 School of Public Health, Li Ka Shing Faculty of Medicine, The University of Hong Kong, G/F, Patrick Manson Building (North Wing), 7 Sassoon Road, Pok Fu Lam, Hong Kong

**Keywords:** Cervical Cancer, Magnetic resonance imaging, Diffusion-weighted imaging, Intravoxel incoherent motion, Perfusion, Lymph node metastasis

## Abstract

**Background:**

Magnetic resonance imaging (MRI) has limited accuracy in detecting pelvic lymph node (PLN) metastasis. This study aimed to examine the use of intravoxel incoherent motion (IVIM) in classifying pelvic lymph node (PLN) involvement in cervical cancer patients.

**Methods:**

Fifty cervical cancer patients with pre-treatment magnetic resonance imaging (MRI) were examined for PLN involvement by one subspecialist and one non-subspecialist radiologist. PLN status was confirmed by positron emission tomography or histology. The tumours were then segmented by both radiologists. Kruskal-Wallis tests were used to test for differences between diffusion tumour volume (DTV), apparent diffusion coefficient (ADC), pure diffusion coefficient (D), and perfusion fraction (*f*) in patients with no malignant PLN involvement, those with sub-centimetre and size-significant PLN metastases. These parameters were then considered as classifiers for PLN involvement, and were compared with the accuracies of radiologists.

**Results:**

Twenty-one patients had PLN involvement of which 10 had sub-centimetre metastatic PLNs. DTV increased (*p* = 0.013) while ADC (*p* = 0.015), and *f* (*p* = 0.006) decreased as the nodal status progressed from no malignant involvement to sub-centimetre and then size-significant PLN metastases. In determining PLN involvement, a classification model (DTV + *f*) had similar accuracies (80%) as the non-subspecialist (76%; *p* = 0.73) and subspecialist (90%; *p* = 0.31). However, in identifying patients with sub-centimetre PLN metastasis, the model had higher accuracy (90%) than the non-subspecialist (30%; *p* = 0.01) but had similar accuracy with the subspecialist (90%, *p* = 1.00). Interobserver variability in tumour delineation did not significantly affect the performance of the classification model.

**Conclusion:**

IVIM is useful in determining PLN involvement but the added value decreases with reader experience.

## Background

The inclusion of PLN metastatic status into the recently revised International Federation of Gynaecology and Oncology (FIGO) staging system has shown to be prognostic on historical cohort, hence may better identify patients for radiation dose escalation to suspicious PLNs to improve locoregional control and survival [1–4]. In cervical cancer, MRI is used to evaluate the primary tumour extent and at the same time to assess lymph node involvement, in which the diagnosis of the latter relies on size- and morphology-based criteria on T2-weighted (T2W) images [5–7]. However, these criteria have shortcomings in that they are susceptible to false positives due inflammatory enlarged lymph nodes [8, 9] and false negatives due to difficulties in detecting sub-centimetre metastatic PLNs [10]. Ultimately, these criteria have resulted in low pooled sensitivity of 53–56% despite high specificity of 91–93% [11, 12].

Pathological assessment of resected lymph nodes provides definitive diagnosis; however, pelvic lymph node dissection (PLND) is not routinely performed in early bulky and locally advanced cervical cancer (LACC) treated by concurrent chemoradiation (CCRT) [13]. FDG-PET/CT, which has been shown to have high accuracy [11] in identifying PLN involvement, is used clinically to determine the nodal status but imparts substantial radiation burden with its high cost and limited availability. Therefore, it can be useful in identifying non-invasive imaging features of the primary tumour on MRI that are associated with the presence of PLN metastases in cervical cancer.

There has been interest in the use of diffusion-weighted imaging (DWI) and apparent diffusion coefficient (ADC) to characterize the primary tumour in cervical cancer, and ADC has been shown to be associated with various clinicopathological factors such as FIGO stage, histological grade, including nodal status [14–16]. Previous studies have demonstrated the utility of ADC to distinguish between benign and malignant mediastinal masses as well as axillary lymph nodes in patients with breast cancer [17, 18]. However, the diffusional signal in cervical cancer is thought to be better ascribed to the intravoxel incoherent motion (IVIM) biexponential model and the perfusion effects are non-negligible [19–21]. There is growing interest in applying advanced diffusion models, such as IVIM, diffusion kurtosis imaging (DKI), and diffusion tensor imaging (DTI) to discriminate between malignant and benign masses as well as characterize tumours [22–24]. Specifically in the female pelvis, previous studies have demonstrated the feasibility of IVIM and suggested that this technique could be used to aid in the clinical management of LACC patients [20, 25, 26].

The purpose of this study was to assess the classification performances of IVIM parameters of the primary cervical tumour in classifying PLN involvement, particularly in the detection of sub-centimetre lymph node metastasis, and to compare them with lymph node staging performance of radiologists via visual assessment.

## Methods

### Patients

This retrospective study was approved by and done in accordance with the regulations set by the local Institutional Review Board. This study involved anonymized human data without identifying information that has already been collected waiving the need for informed consent. Patients were retrospectively recruited from March 2012 to January 2018. Inclusion criteria were: histologically confirmed squamous cell carcinoma (SCC) or adenocarcinoma LACC; FIGO stage of IB2 or higher; treatment-naïve; and who underwent either additional pre-operative FDG-PET/CT staging or subsequent PLND. A total of 50 consecutive patients were thus identified.

### MRI

Patients were asked to fast at least 6 h before the examination and 20 mg hyoscine butylbromide (Buscopan, Boehringer Ingelheim, Germany) was given intramuscularly at the beginning of each examination to reduce bowel peristalsis. Images were acquired with a 3 T MRI system (Achieva 3.0 T TX, Philips Healthcare, Best, the Netherlands) using a dedicated 16-channel phased array torso coil. All patients were imaged on the same scanner. The conventional sequences are tabulated on Table 1. DWI utilized single-shot spin-echo echo-planar imaging, which was acquired immediately after the axial T2W imaging. It was performed in free breathing with background body signal suppression (pre-saturation inversion recovery fat suppression) with parallel imaging and sensitivity encoding (SENSE) factor of 2. Thirteen *b*-values (0, 10, 20, 30, 40, 50, 75, 100, 150, 300, 500, 800, and 1000 s/mm^2^) in the axial plane encompassing 20 slices to include the entire primary tumour motion-probing gradients in three orthogonal axes.

**Table 1:** Summary of MRI scan parameters. CE: contrast-enhanced, DWI: diffusion-weighted imaging; FFE: fast field echo; TR/TE: repetition time/echo; TSE: turbo spin echo; SPAIR: Spectral Attenuation Inversion Recovery; SENSE: sensitivity encoding

Sequences	T2W TSE	T2W SPAIR	T2W TSE	DWI	CE 3D T1W FFE
Plane	Sagittal	Coronal	Axial	Axial	3D
TR/TE (ms)	4000/80	3500/80	2800/100	2000/54	3/1.4
Turbo factor	30	21	12	NA	NA
SENSE factor	2	2	2	2	2
Field of view (mm)	240 × 240	230 × 230	402 × 300	406 × 300	370 × 203
Matrix size	480 × 298	352 × 300	787 × 600	168 × 124	248 × 134
Slice thickness (mm)	4	4	4	4	1.5
Intersection gap (mm)	0	0	0	0	0
Bandwidth (Hz/pixel)	230	186	169	15.3	724
Number of excitations	2	1	1	2	1

### Image analysis

**Fig. 1:**
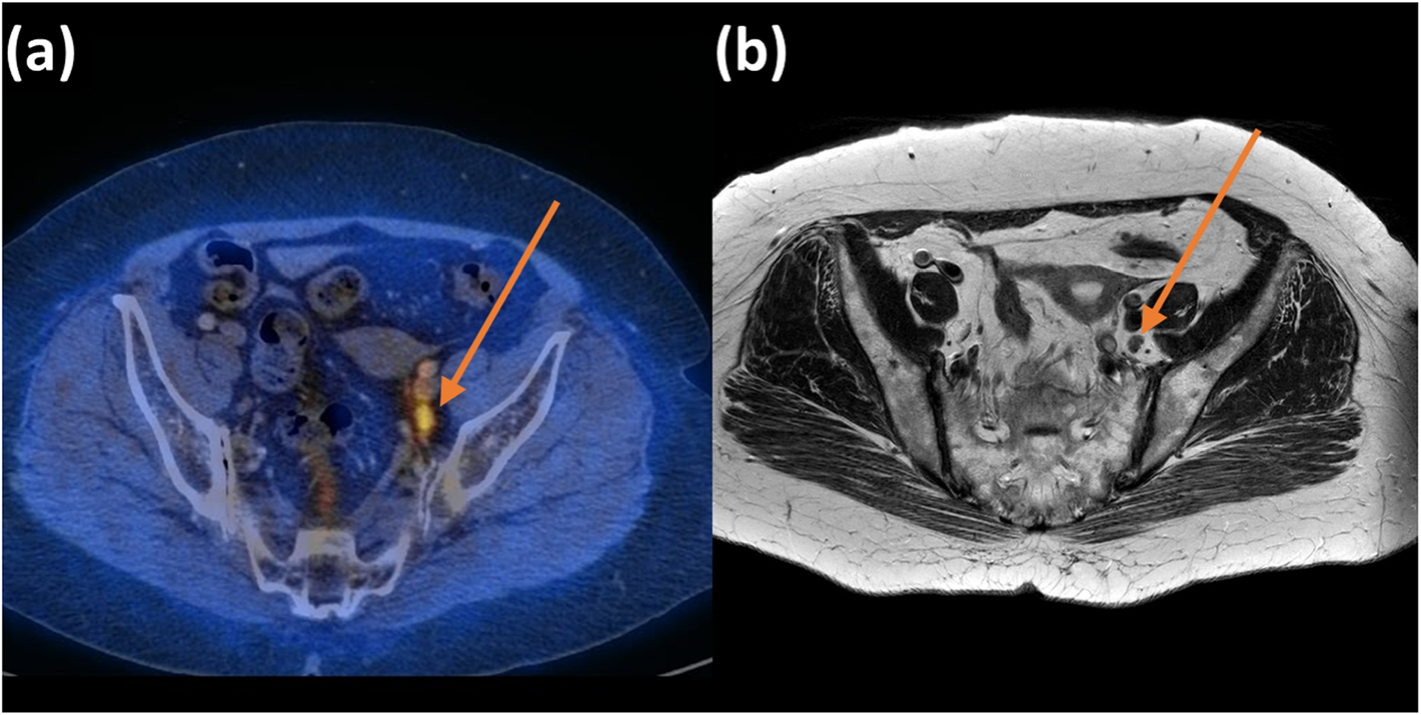
A case of a 69-year-old patient International Federation of Gynaecology and Obstetrics (FIGO) staged IIA with a sub-centimetre pelvic lymph nodes on parametric maps of (**a**) axial fused ^18^F-fluoro-deoxyglucose positron emission tomography and computed tomography (FDG-PET/CT) image and (**b**) axial T2-weighted (T2W) image. This patient was classified as metastatic by the IVIM models and correctly staged by the subspecialist but not the non-subspecialist.

**Fig. 2:**
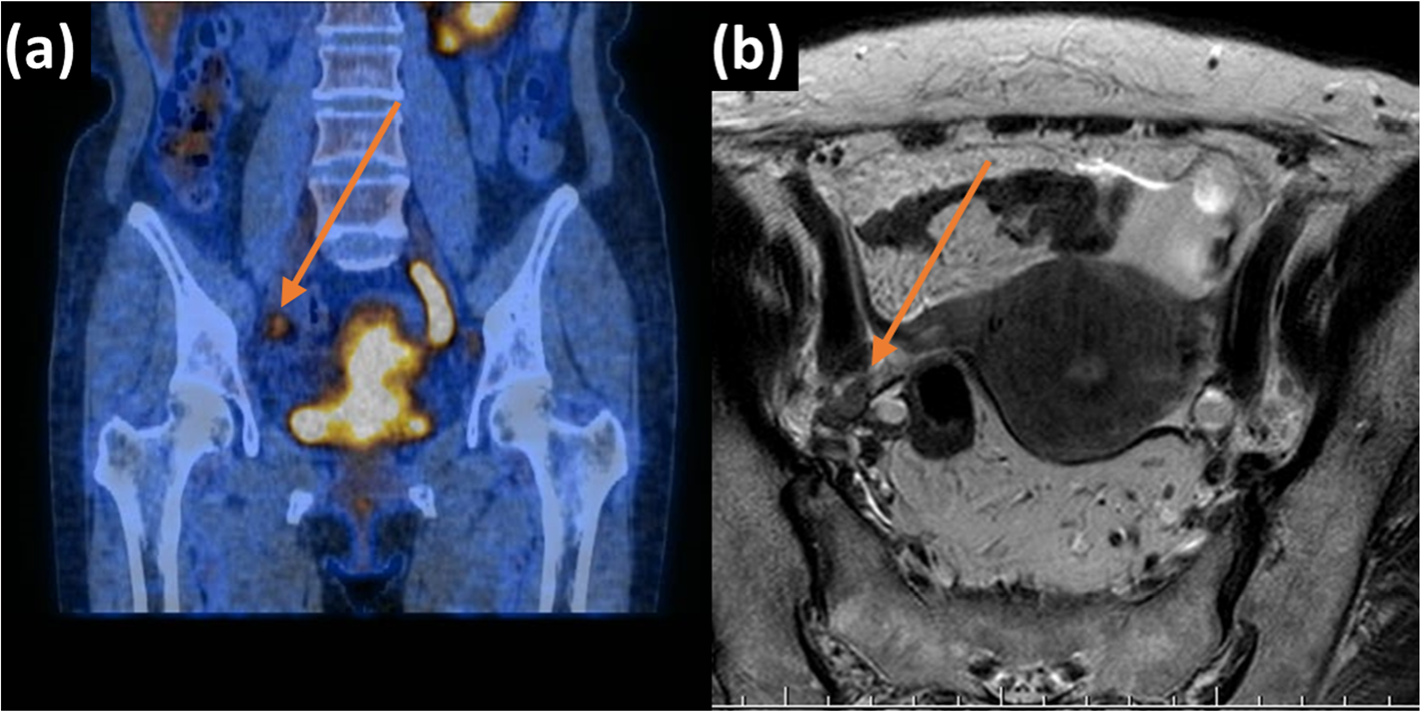
A case of a 67-year-old patient International Federation of Gynaecology and Obstetrics (FIGO) staged IIIB with a sub-centimetre pelvic lymph node on parametric maps of (**a**) coronal fused ^18^F-fluoro-deoxyglucose positron emission tomography and computed tomography (FDG-PET/CT) image and (**b**) axial T2-weighted image. This patient was classified as metastatic by the IVIM models and correctly staged by the subspecialist but not the non-subspecialist.

Two board-certified radiologists, one subspecialist in gynae-oncological imaging (> 10 years of experience in female pelvic imaging and cross-sectional imaging) and one non-subspecialist (> 10 years of experience in cross-sectional imaging), staged patients’ PLN status by visually examining for PLN involvement using the size, morphology and signal-based criteria on T2W images and DWI [5–7]. In short, PLNs with short axis larger than 1 cm on T2W-MRI, round morphology and/or containing signal similar to the primary tumour was considered malignant. PLN involvement was then confirmed using FDG-avidity from pre-operative staging FDG-PET/CT or subsequent histological findings from PLND (Figs. 1 and 2). Any FDG uptake more than the background liver activity along the pelvic nodal chain was considered positive [27].

**Fig. 3:**
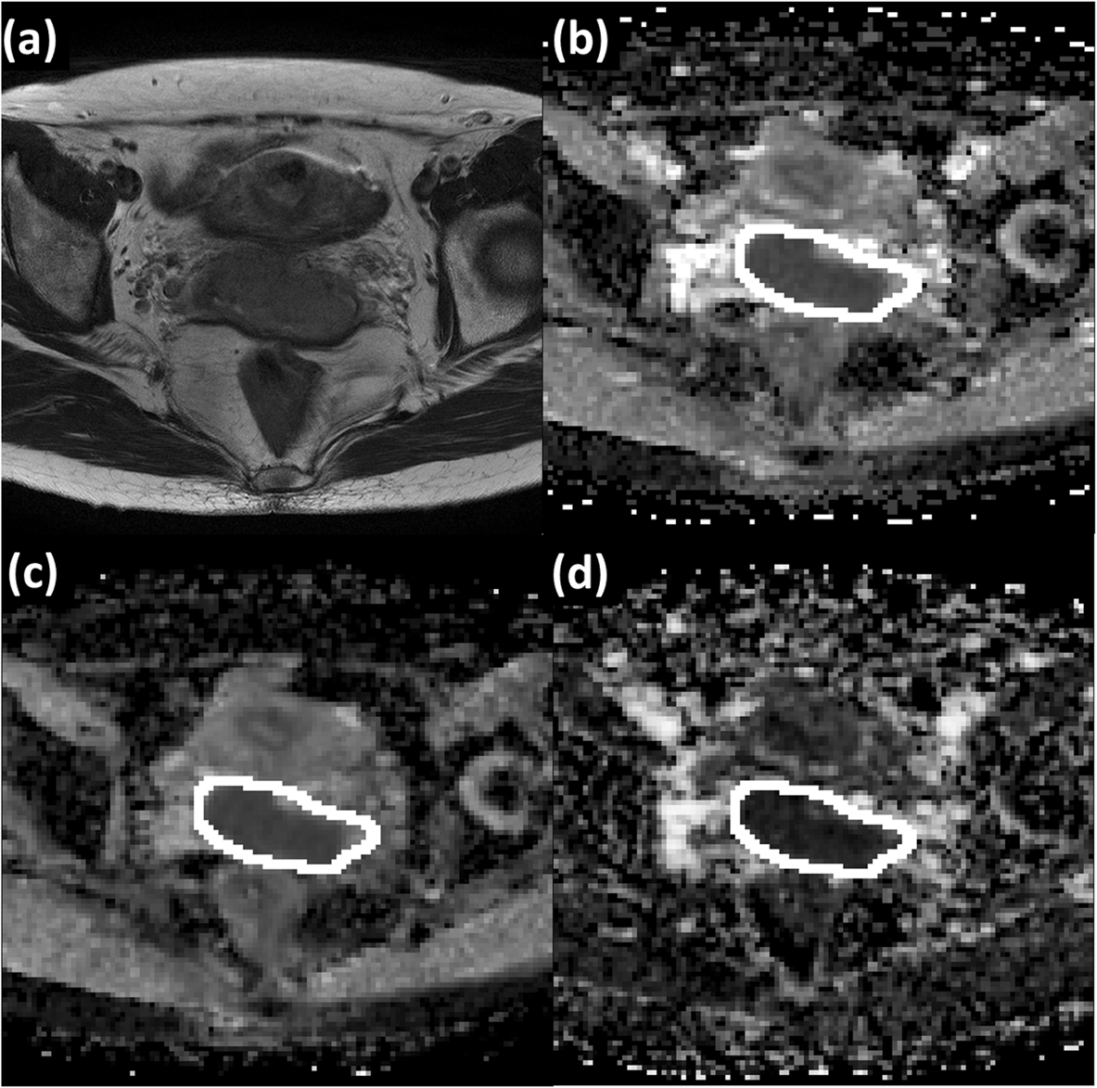
Representative example of the placement of region of interest in the parametric maps of (**a**) T2-weighted image, (**b**) apparent diffusion coefficient (ADC), (**c**) pure diffusion coefficient (D), and (**d**) perfusion fraction (*f*) on a case of a 66-year old patient International Federation of Gynaecology and Obstetrics (FIGO) staged IIA2. This was repeated on subsequent slices to include the entire tumour volume.

Volumes of interest (VOIs) were manually drawn by both radiologists to encompass the entirety of primary tumours on the *b*1000 images with reference to co-registered T2W images and ADC maps. Areas of hyperintensity on the *b*1000 maps were taken as the primary tumour and radiologists segmented the tumour with reference to co-registered T2W images and ADC maps. These VOIs were then copied to co-registered parametric maps. IVIM maps were visually inspected and VOIs were adjusted if needed (Fig. 3).

### DWI analysis

The monoexponential model of DWI was based on the geometric averaged DWI signal from three orthogonal axes. ADC map was calculated with 2 *b*-values (0, 1000 s/mm^2^) using the function:

1 $$ \frac{S_b}{S_0}=\exp \left(-b\bullet \mathrm{ADC}\right) $$

where S_b_ represents the mean signal intensity with the diffusion gradient, *b*, S_0_ is the mean signal intensity when *b* = 0 s/mm^2^. Diffusion tumour volume (DTV) was calculated by multiplying the tumour areas on *b*1000 images by the slice thickness.

The biexponential model, IVIM, was analysed using all 13 *b*-values acquired and parametric maps of D, *f*, and pseudo-diffusion coefficient (D*) were generated using the biexponential model described by the function:

2 $$ \frac{S_b}{S_0}=f\exp \left(-b\left(D+{D}^{\ast}\right)\right)+\left(1-f\right)\exp \left(-b\bullet \mathrm{D}\right) $$

A 3 × 3 gaussian smoothing filter was first applied for pre-processing before fitting using non-linear least squares with non-negative constraints under a Levenberg-Marquardt routine by an in-house algorithm in MATLAB (The MathWorks Inc., Natick, MA, USA). The two-step approach was implemented by estimating D first, then followed by *f* and D*. Incorrectly fitted pixels with pixel values of ADC and D > 3 × 10^− 3^ mm^2^/s, and *f* > 1 were excluded from analysis [28, 29]. Mean values of the parameters ADC, D, and *f* were then calculated and considered for subsequent analysis. D* was excluded from further analysis due to low signal-to-noise ratio [28, 30].

### Statistical analysis

All statistical analyses were executed in R version 3.2.3 (R Development Core Team).

#### Primary tumour associations with nodal status

The cohort was stratified into three groups: patients with no nodal involvement, patients with nodal involvement but whose largest PLN had a short axis of less than 1 cm on T2W images (sub-centimetre involvement), and patients with at least one PLN larger than 1 cm (size-significant involvement). Kruskal-Wallis tests were then used to test for ADC and IVIM parameter differences between the nodal involvement groups and Nemenyi tests for pairwise comparisons between groups.

The Fisher’s exact test was used to test for associations between PLN involvement with histological sub-type and with FIGO stage.

#### Interobserver and intraobserver reproducibility

Intraclass Correlation Coefficient (ICC) analysis was used to assess interobserver variability of ADC and IVIM parameters quantified from the VOIs of both radiologists. ICC measures agreement and ranges from 0 to 1, where values between 0.50–0.75 are considered moderate, 0.76–0.90 are considered good, and above 0.90 are considered excellent agreement [31].

#### Classification models of PLN staging

A logistic regression model based on IVIM histogram features derived from the VOIs of the subspecialist was developed to classify PLN involvement (regardless of PLN size) where up to 2 features were selected [32]. Feature selection was executed using stepwise forward regression. To test for the effect of interobserver variation in manual segmentation, the developed model was fitted on features derived from the VOIs of the non-subspecialist.

Model performance were compared to the performances of the radiologists’ nodal staging. The models were assessed using receiver operating characteristic (ROC) analysis. Relative performance of the models was assessed by computing the Z-statistic of the accuracy confidence intervals of each model’s accuracy, i.e. the degree of CI overlap between of two models’ accuracies, using the IVIM classification model based on the VOIs of the subspecialist as reference [33].

## Results

### Clinicopathological characteristics

The median age of patients was 54 (28–78) years old. Thirty-five patients were of the SCC sub-type and the remaining 15 were of the adenocarcinoma sub-type. Eighteen patients were FIGO stage IB, 5 were FIGO stage IIA, 10 were stage IIB, 2 were FIGO stage IIIA, 14 were FIGO stage IIIB, and 1 was FIGO stage IVB. There was an average of 12.8 days between the MRI scan and FDG-PET/CT or PLND. Pelvic lymph node assessment by FDG-PET/CT was done in 41 patients, while 9 patients had PLND. Twenty patients were found to have PLN involvement by FDG-PET/CT assessment. One PLND candidate was found to have PLN involvement. Of the 21 patients with PLN involvement, 10 had sub-centimetre involvement. The mean DTV was 37.68 (0.95–193.58) cm^3^. There were no associations between PLN involvement with histological sub-type (*p =* 0.074) and FIGO staging (*p* = 0.254).

### Tumour characteristics

**Fig. 4:**
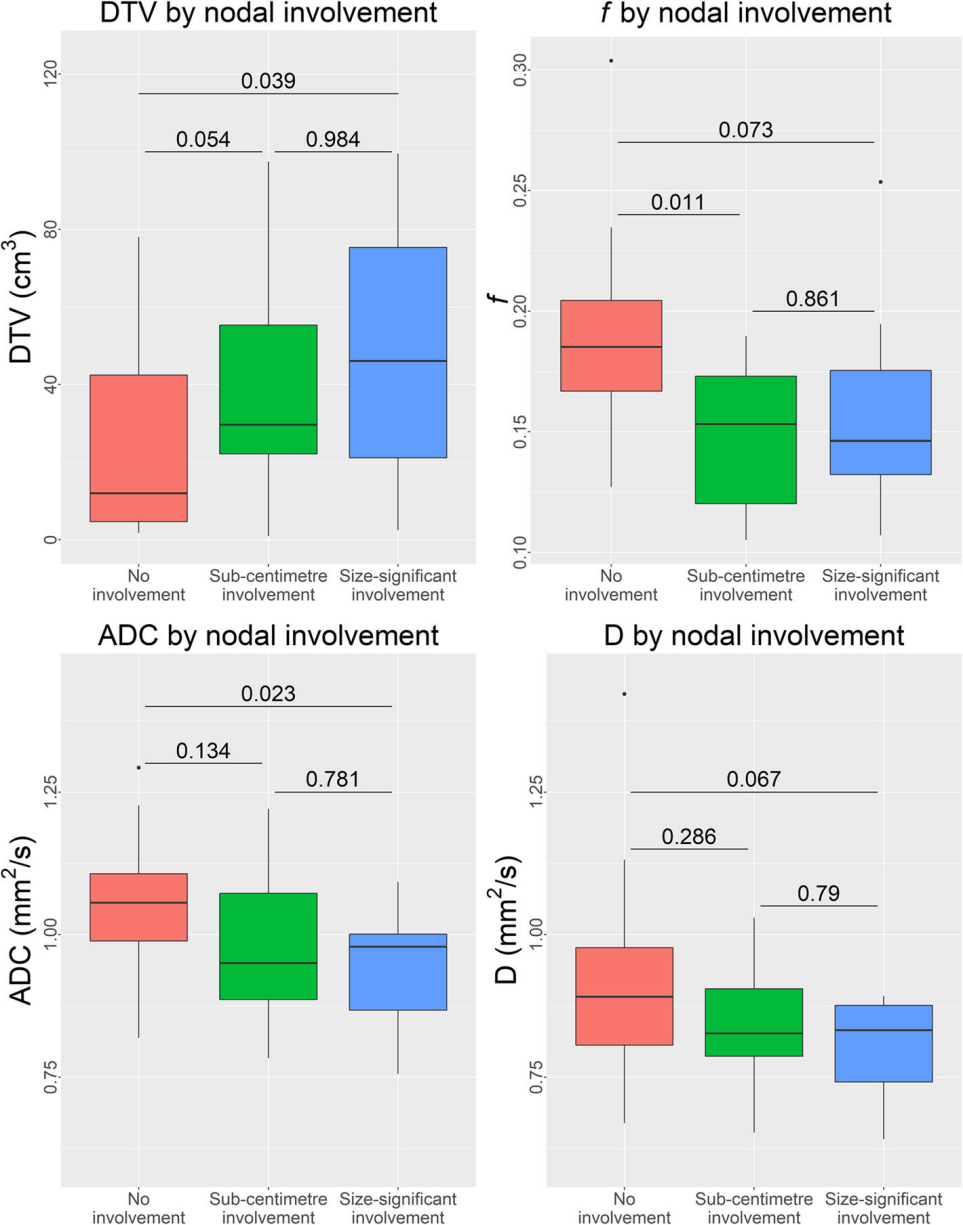
Boxplots of the diffusion tumour volume (DTV), perfusion fraction (*f*), apparent diffusion coefficient (ADC), and pure diffusion coefficient (D) measurements of the primary tumour separated by nodal involvement with pairwise group comparisons.

DTV increased while ADC, D, and *f* decreased as the nodal status progressed from no malignant involvement to sub-centimetre and then size-significant PLN involvement, though the differences in D among the different groups were not significant (Table 2 and Fig. 4). Pairwise group comparisons may be found on Fig. 4. Interobserver variability of ADC, D, and *f*, were excellent (ICC = 0.961, 0.947, and 0.943 respectively).

**Table 2:** Apparent diffusion coefficient (ADC) and intravoxel incoherent motion (IVIM) parameter values of the primary tumour between patients without nodal involvement, those with sub-centimetre involvement, and those with size-significant involvement. ADC: apparent diffusion coefficient (x 10^− 3^ mm^2^/s); D: pure diffusion coefficient (x 10^− 3^ mm^2^/s); *f*: perfusion fraction. PLN: pelvic lymph node

	No involvement	Sub-centimetre involvement	Size-significant involvement	***p***-value
DTV (cm^3^)	22.84 ± 22.84	54.04 ± 52.42	50.18 ± 34.63	0.013
ADC (10^−3^ mm^2^/s)	1.07 ± 0.15	0.98 ± 0.14	0.93 ± 0.11	0.015
D (10^− 3^ mm^2^/s)	0.91 ± 0.16	0.84 ± 0.12	0.80 ± 0.09	0.057
*f*	0.19 ± 0.04	0.15 ± 0.03	0.16 ± 0.04	0.006

### PLN staging

Tabulated diagnostic performances of radiologists may be found in Table 3. The optimal IVIM classification model consisted of DTV and *f*. Classification accuracy of the IVIM classification model to determine PLN involvement regardless of size was similar to the diagnostic accuracies of subspecialist and non-subspecialist (Table 3). However, the IVIM model correctly identified 9 of the 10 patients with sub-centimetre PLN involvement, which gave it the same performance as the subspecialist (*p* = 1.00) but better performance that the non-subspecialist (*p* = 0.01) (Table 4). The performance of the classification model was not susceptible to interobserver variation in the manual segmentation of the primary tumour.

**Table 3:** Pelvic lymph node involvement classification performances of the radiologists as well as the classification performances of the intravoxel incoherent motion (IVIM) classification models. The *p*-values of the relative classification performances of each model are given where the first IVIM classification model served as the reference. The first IVIM model used parameters dervied from the subspecialist’s tumours segmentations, and the second IVIM model used parameters dervied from the non-subspecialist’s tumour segementations. VOI: Volume of Interest

		Accuracy	Sensitivity	Specificity	***p-value***
**Radiologists**	Subspecialist	0.90	0.95	0.86	0.31
	Non-subspecialist	0.76	0.62	0.86	0.73
**Model**	Subspecialist VOI	0.80	0.71	0.86	ref
	Non-subspecialist VOI	0.82	0.80	0.82	0.86

**Table 4:** Patients with sub-centimetre metastatic pelvic lymph node (PLN) involvement and the nodal staging given by the radiologists as well as the PLN classification given by the intravoxel incoherent motion (IVIM) models. Correct staging or classification of PLN involvement despite size-insignificance are marked with the character ‘X’

Code	Subspecialist	Non-subspecialist	IVIM Model (Subspecialist)	IVIM Model (Non-subspecialist)
1	X		X	X
13	X		X	X
17	X		X	X
21	X		X	X
23	X		X	X
28	X		X	X
30	X	X	X	X
32		X		
38	X	X	X	X
44	X		X	X

## Discussion

In this current study, we demonstrated that DTV increased while ADC, D, and *f* decreased in the primary tumour as the nodal status progressed from no malignant involvement to sub-centimetre and then size-significant PLN metastases. The accuracy of the IVIM classification model was comparable to radiologists in classifying PLN involvement regardless of size but it had higher accuracy compared to the non-subspecialist in the detection of sub-centimetre metastatic PLNs.

The decreasing trend in ADC observed in this study suggests that more invasive tumours are characterized by increased cellularity. This was in corroboration with the previous study by *Schob* et al. which found that node-positive tumours had significantly lower ADC compared to node-negative tumours [34], though a study by *Xue* et al. found no difference in ADC values between the two groups [35]. The discrepancy may be due to the differences in DWI acquisition, in which the former had a similar acquisition to the present study (3 T MRI with b-values 0 and 1000 s/mm^2^), while the latter used a lower field strength and different b-value combination (1.5 T MRI with b-values 0 and 800 s/mm^2^).

We also observed a decreasing trend in D, though this trend was not significant, which suggests that there are some non-negligible perfusion effects. Immunohistochemical studies have suggested that acute hypoxia from reduced tumour perfusion increased the tumour’s metastatic capacity [36, 37]. Similarly, dynamic contrast-enhanced (DCE) MRI studies have shown that poor perfusion was associated with an increase in the nodal metastatic capacity of the primary tumour [37, 38], which could be related to the poor tumour oxygenation [39, 40]. While it is believed that the two techniques do not measure the same perfusion phenomenon [41, 42], the study by *Lee* et al. found significant correlations between IVIM perfusion and DCE perfusion in cervical cancer [43]. Moreover, a previous study found that dynamic susceptibility contrast (DSC) percentages were lower in malignant cervical nodes compared to benign nodes which indicate restricted microcapillary perfusion [44]. This may explain why the perfusivity characteristics, rather than microarchitecture of the primary tumour, adds value in classifying the metastatic propensity in cervical cancer.

It has been shown that at least part of a PLNs has similar microarchitecture to the primary tumour [45]. We observed significant differences in ADC and *f* values of the primary tumour between PLN involvement groups, but not in D percentiles. Our observation partially concurred with the study by *Wu* et al. that found that metastatic LNs had lower *f* values; however, they found that PLNs had higher D values [46]. In that study, lymph nodes with small volumes were included in analysis and might affect quantification of diffusion parameters due to partial volume effects [47], potentially accounting for the discrepant result observed in our study. On the other hand, the observed ADC results concur with the other studies that attributed lower ADC in metastatic lymph nodes to tumour tissue invasion, leading to increased cellularity and enlarged cell size [48–50].

The IVIM classification model suggests that PLN involvement is best characterised by the tumour’s diffusion volume and perfusivity and its overall accuracy was similar to that of radiologists. When identifying patients with sub-centimetre PLN involvement, the subspecialist performed better than the non-subspecialist, likely related to the greater experience and more emphasis placed on the morphology and signal of the PLN over the size criterion. The IVIM classification model had similar accuracy as the subspecialist’s staging in identifying patients with sub-centimetre metastatic PLNs. As we did not observe any significant differences in any parameters examined between tumours with sub-centimetre involvement and those with size significant involvement, it is likely that these tumours have similar diffusion and perfusion characteristics. This suggests that parametric IVIM could serve as adjunct tool for a non-subspecialist and provide an objective quantification when staging lymph node involvement.

This study has several limitations. First, this was a retrospective, single-centre study with a relatively small sample size. Second, extended scan coverage and the direct measurements of the diffusional signals of PLNs and were not performed due to the inherently longer scan time with IVIM. Though not clinically routine, previous studies have explored use of quantitative DWI to detect lymph node metastasis, which was shown to have a pooled sensitivity of 86–87% and specificity of 83–84% [12, 51]. Third, while the existence hyperplastic lymph nodes has been noted as a major source of false positives in MRI PLN assessment, we could not analyse hyperplastic lymph nodes due to the low number of false positives in our radiologists’ classifications. Lastly, only a small proportion of our patients (*n =* 9) had histological confirmation of the nodal status and majority (*n =* 41) had their nodal status determined by FDG-PET/CT. FDG-PET/CT is supported by the latest FIGO staging guidelines in nodal assessment given its high sensitivity of 83%, especially in PLN staging and low false negative rate of 4–15% in LACC [12, 52, 53].

## Conclusions

IVIM analysis of the primary tumour in cervical cancer is potentially useful in determining PLN involvement and could provide more objective and quantifiable evaluation, but its added value is diminished as reader experience increases in the detection of sub-centimetre PLNs.

## Data Availability

Please contact the corresponding author regarding any requests for the data used or analysed in this study.

## Abbreviations

ADC: Apparent Diffusion Coefficient
CI: Confidence Interval
CCRT: Concurrent Chemoradiotherapy
D: Pure Diffusion Coefficient
DCE: Dynamic Contrast Enhanced
DKI: Diffusion Kurtosis Imaging
DTI: Diffusion Tensor Imaging
DTV: Diffusion Tumour Volume
DSC: Dynamic Susceptibility Contrast
DWI: Diffusion Weighted Imaging
*f*: Perfusion fraction
FDG-PET/CT: ^18^F-fluoro-deoxyglucose Positron Emission Tomography Combined with Computed Tomography
FIGO: International Federation of Gynaecology and Oncology
ICC: Intraclass Correlation Coefficient
IVIM: Intravoxel Incoherent Motion
LACC: Locally Advanced Cervical Cancer
MRI: Magnetic Resonance Imaging
PLN: Pelvic Lymph Node
PLND: Pelvic Lymph Node Dissection
ROC: Receiver Operating Characteristic
SCC: Squamous Cell Carcinoma
T2W: T2-Weighted
VOI: Volumetric Region of Interest
